# The Natural History of Post-Chikungunya Viral Arthritis Disease Activity and T-cell Immunology: A Cohort Study

**DOI:** 10.33696/immunology.6.191

**Published:** 2024

**Authors:** Aileen Yu-hen Chang, Alfonso Sucerquia Hernández, Jose Forero Mejía, Sarah Renee Tritsch, Evelyn Mendoza-Torres, Liliana Encinales, Andres Cadena Bonfanti, Abigale Marie Proctor, Gary Leonard Simon, Samuel Joseph Simmens, Gary Steven Firestein

**Affiliations:** 1 Department of Medicine, George Washington University, Washington, DC, USA; 2 Department of Global Health, Milken Institute School of Public Health, George Washington University, Washington, DC, USA; 3 Advanced Biomedicine Research Group, Universidad Libre de Colombia, Seccional Barranquilla, Barranquilla, Atlántico, Colombia; 4 Department of Medicine, Allied Research Society, Barranquilla, Atlántico, Colombia; 5 Centro de Investigación, Clínica de la Costa SAS, Barranquilla, Atlántico, Colombia; 6 Department of Biostatistics and Bioinformatics, Milken Institute School of Public Health, George Washington University, Washington, DC, USA; 7 Department of Medicine, UC San Diego School of Medicine, San Diego, CA, USA

**Keywords:** Chikungunya virus, Arthritis, Arthritis disease severity, T-cell populations, Disability, Pain, Stiffness

## Abstract

**Background:**

Chikungunya virus (CHIKV) is an alphavirus spread by mosquitos that causes arthralgias and arthritis that may last for years. The objective of this study was to describe the arthritis progression and T cell immunology over a two-year period.

**Methods:**

A cohort of 40 cases of serologically confirmed CHIKV from Magdalena and Atlántico, Colombia were followed in 2019 and again in 2021. Arthritis disease severity, disability, pain, stiffness, physical function, mobility, fatigue, anxiety, sleep disturbances and depression were assessed. Serum cytokines and T-cell subsets were measured and tested for change. Correlations within each of the 2 time periods for laboratory parameters were also examined.

**Results:**

Although, arthritis disease severity, as measured by the Disease Activity Score-28 (DAS-28) did not change significantly over a two-year period, a new metric- the Chikungunya Disease Activity Score (CHIK-DAS)- was more sensitive to detect changes in disease severity than the Disease Activity Score-28 (DAS-28) and showed some improvement in average disease severity from moderate to mild over two years. Cases were characterized by moderate disability, pain, and stiffness with mild alterations of physical function, mobility, fatigue, anxiety, sleep disturbances and depression that did not change significantly over time. Small joints including the fingers and wrists were most affected without significant change over time. The percentage of effector T cells (Teffs) and regulatory T cells (Tregs) of CD4^+^ T cells both decreased over time. Teff percentages decreased more significantly resulting in a halving of the Teff/Treg ratio two years later. Furthermore, markers of Treg immunosuppressive function such as CTLA4, Helios, CD28, CD45RA and 41bb decreased over time. Cytokines did not change significantly over time.

**Conclusions:**

The presented data suggest that arthritis persists almost seven years after chikungunya infection in some patients with waning Teff and Treg numbers and activation markers over time. Treg activation may be a promising therapeutic target for further investigation.

## Introduction

Chikungunya virus (CHIKV) is an alphavirus spread by mosquitos that causes arthralgias and arthritis that may last for months to years [1,2]. The name chikungunya comes from the Makonde language and means “bent over in pain” [3]. The Pan American Health Organization reported 410,017 cases of chikungunya in sixteen countries in the Americas in 2023 [4]. Recent large outbreaks have affected Brazil (264,767 cases), Paraguay (140,905 cases) and Bolivia (1,468) in 2023 [4]. CHIKV circulates in 115 countries globally and incidence of infection is increasing due to factors such as unplanned urbanization and climate change that affect mosquito and human population dynamics [3]. Little is known about the disease course of arthritis initiated by chikungunya viral infection multiple years after initial infection as few cohorts are followed for extended durations [5]. While the arthralgias improve progressively over time with most patients [5], the clinical characteristics of those patients who have persistent arthritis, the underlying immunology and how to effectively treat them is largely unknown.

Rheumatoid arthritis medications are often repurposed for the persistent arthritis caused by CHIKV, but targeted medications are lacking. The French and Brazilian guidelines suggests that those with persistent arthritis years after the initial infection have an autoimmune arthritis that may respond to disease modifying anti-rheumatic drugs (DMARDS) however randomized placebo controlled trials are lacking [6,7]. T-cells are an important potential target as T-cells are obligatory for the development of arthritis in CHIKV mouse models [8]. This cohort study describes the patient demographics and 2- year disease course of forty patients with CHIKV arthritis four to six years after initial infection highlighting changes in T-cell subsets over time.

## Material and Methods

### Study Design

Cases of clinically and serologically confirmed CHIKV infection age 18 and over from Magdalena and Atlántico Departments, Colombia were enrolled and followed as a part of the CHIKV cohort. Participants had their first visit in November 2019 and a follow up visit in August 2021. An in-person history and physical were conducted to ascertain demographic characteristics, exposure history, and arthritis signs and symptoms. Blood samples were collected to determine levels of serum cytokines and T-cell subsets.

### Ethics Statement

The study protocol was approved by The George Washington University Institutional Review Board (Protocol: Colombian Arboviral Surveillance Protocol IRB #121611, GWU IRB, Washington, D.C., USA (FWA00005945) and the Clinica de la Costa IRB, Colombia (FWA IORG0008529)). Research on human subjects was conducted in compliance with regulations relating to the protection of human subjects and adhered to principles identified in the Belmont Report (1979). All data collection and research on human subjects for this publication were conducted under an IRB-approved protocol. All participants were adults and provided written informed consent during an in-person interview.

### Measures

Disease activity was assessed using the Disease Activity Score (DAS)-28 [9] with C-reactive protein (CRP), which includes assessment of the number of swollen and tender joints (out of the 28), CRP, and a visual analog global assessment of health. A DAS-28 of less than 2.6 indicates remission, 2.6-3.2 indicates low disease activity, 3.2-5.1 indicates moderate disease activity, and greater than 5.1 implies severe disease activity.

The chikungunya disease activity score (CHIK-DAS) is a chikungunya specific measure of arthritis disease activity that includes the DAS-28 with the addition of ankle tenderness and a stiffness item that are prominent components of chikungunya arthritis (publication pending). In comparison to the DAS-28, the CHIK-DAS had improved predictive value for a composite outcome of disability, pain, physical and mental quality of life and mobility. Disease activity cutoffs include remission (<40), mild (40-49.99), moderate (50-59.99) and severe (60+) disease. The CHIK-DAS was also assessed.

The Health Assessment Questionnaire (HAQ) Disability Index [10] was used to measure physical function, composed of a four-level difficulty scale for each item that is scored from 0 to 3, representing normal/no difficulty (0), some difficulty (1), much difficulty (2), and unable to do (3). There are 20 questions in eight categories of functioning – dressing, rising, eating, walking, hygiene, reach, grip, and usual activities. The value of the HAQ index can be interpreted in terms of three categories: mild difficulties to moderate disability (0–1), moderate to severe disability (1–2), and severe to very severe disability (2–3). Disability measured by the HAQ has repeatedly been correlated with mortality rates, progression of aging, and healthcare resource utilization.

Measures of stiffness and pain are of specific importance to quantifying CHIKV arthritis impact as they are associated with multiple domains of quality of life [11]. The Sparra Stiffness Questionnaire is a 12-item score calculated with a range of 0–15 to quantify the burden of stiffness. Pain intensity was assessed on a visual analog scale from 0–100 by both the participant and the physician.

Finally, the Patient-Reported Outcomes Measurement Information System (PROMIS) measures were used to assess quality of life [12]. PROMIS-29 was used to assess five domains (physical function, fatigue, anxiety, sleep disturbance and depression) with four questions for each domain. All domains were evaluated over the previous seven days except for physical function, which has no timeframe specified. Mobility was assessed with a separate four-item questionnaire.

### Sample collection and management

Blood was collected by venipuncture into K2EDTA vacutainers. The blood samples were centrifuged at room temperature (18–25°C) in a horizontal rotor for 20 minutes at 1,500 relative centrifugal force. Plasma was removed from the blood collection tubes and frozen at −80°C until analyzed. PMBCs were cryopreserved after a Ficoll separation.

### Cytokine profiling

Multiplex assessment of a panel of plasma cytokines, including IL-1β, IL-2, IL-4, IL-6, IL-10, IL-12p70, IL-17a, interferon-γ, and tumor necrosis factor (TNF), were measured using the S-PLEX Proinflammatory Human Panel 1 Kit by Meso Scale Diagnostics according to the manufacturer’s instructions.

### T cell measurement via flow cytometry

Flow cytometry was performed on a BD Celesta flow cytometer (BD Biosciences, San Jose, CA) and analyzed in FlowJo 10 (TreeStar Inc., Ashland, OR) to characterize **Tregs** defined as **CD3**^+^**CD4**^+^**CD25**^hi/int+^**CD127**^−^**FoxP3**^+^ and **Teff** defined as **CD3**^+^**CD4**^+^**CD25**^−^**CD127**^−^. All antibodies were titrated prior to use in this study. Peripheral blood mononuclear cells (PBMCs) were isolated from patients as described above and frozen until analysis. Once thawed, PBMCs were washed one time with DMEM containing FBS and once with 1X PBS. T cells were stained in two panels using antibodies against the following markers: CD3, CD4, CD25, CD28, CTLA4, CD127, FoxP3, GARP, Helios, HLA-DR, CD137 (4-1BB), CD45RA, and CCR7 (obtained from Biolegend, San Diego, CA). Live/Dead Fixable Aqua Dead Cell Stain and FoxP3 Fixation/Permeabilization Buffer were obtained from ThermoFisher Scientific (Waltham, MA).

### Data management

All patients were assigned a unique patient identification number, which was used in the database and for labeling patient samples. All patient data were free of personal identifiers and were stored in the REDCap database at The George Washington University.

### CHIKV Serologic confirmation

An immunofluorescence-based assay was used to determine the presence or absence of anti-chikungunya virus IgG antibodies in enrolled patients (Euroimmun, Germany). Plasma samples were used to coat slides fitted with biochips containing chikungunya positive and negative cells. If present in the sample, the IgG antibody reacted with the positive cells and fluoresced. Slides were read using the 488nm excitation laser and a 4x objective on a Biotek Lionheart LX fluorescent microscope. This assay has a sensitivity of 97% and specificity of 96%.

### Sample size and power

Although the study was designed for a larger sample size, recruitment difficulties related to the COVID-19 pandemic limited the sample to 158 participants during 2019, of whom 40 were available to complete a return assessment in 2021. The latter constitutes the sample for this natural history study. Statistical power for detecting change in binary variables will vary by the specific population proportions at the time points. Typical power was approximated by assuming a correlation of r=0.25 between the two study visits. For binary variables, such as treatment received, if the two population proportions are .50 and .22, the resulting power for McNemar’s Test (exact) would be 80%, where alpha=.05 (2-sided). For continuous variables there is 80% power to detect a Cohen’s D effect size of .56 using a paired sample t test. This effect size is often considered “medium” in magnitude.

### Statistical analysis

Frequency distributions and change score distributions were checked visually. Variables were compared between the 2019 visit and the 2021 visit using the paired sample T-test, Wilcoxon Signed-Rank Test or McNemar's Test (exact version) depending on the distribution. Correlations were estimated and tested using the Spearman rank method. Means are presented ± standard deviations. Analyses were conducted using SAS version 9.4 with SAS/STAT 15.3 (SAS Institute Inc).

## Results

### Study sample demographics

The study sample was predominantly female with an average age of 53 ± 15 years, most had at least secondary school education with an average of 4 years since CHIKV infection (Table 1). A comparison of the cohort of participants reported in this analysis with visits in both 2019 and 2021 against participants who had a visit only in 2019 and were lost to follow up is included in Supplementary Tables 1-6. There were no significant differences in demographics, arthritis outcomes, affected joints, medication use, arthritis relapse inciting factors, or c-reactive protein. T-cell subsets had no significant differences except Treg Helios expression was slightly higher in patients only seen in 2019.

### Arthritis characteristics

**Figure 1.**
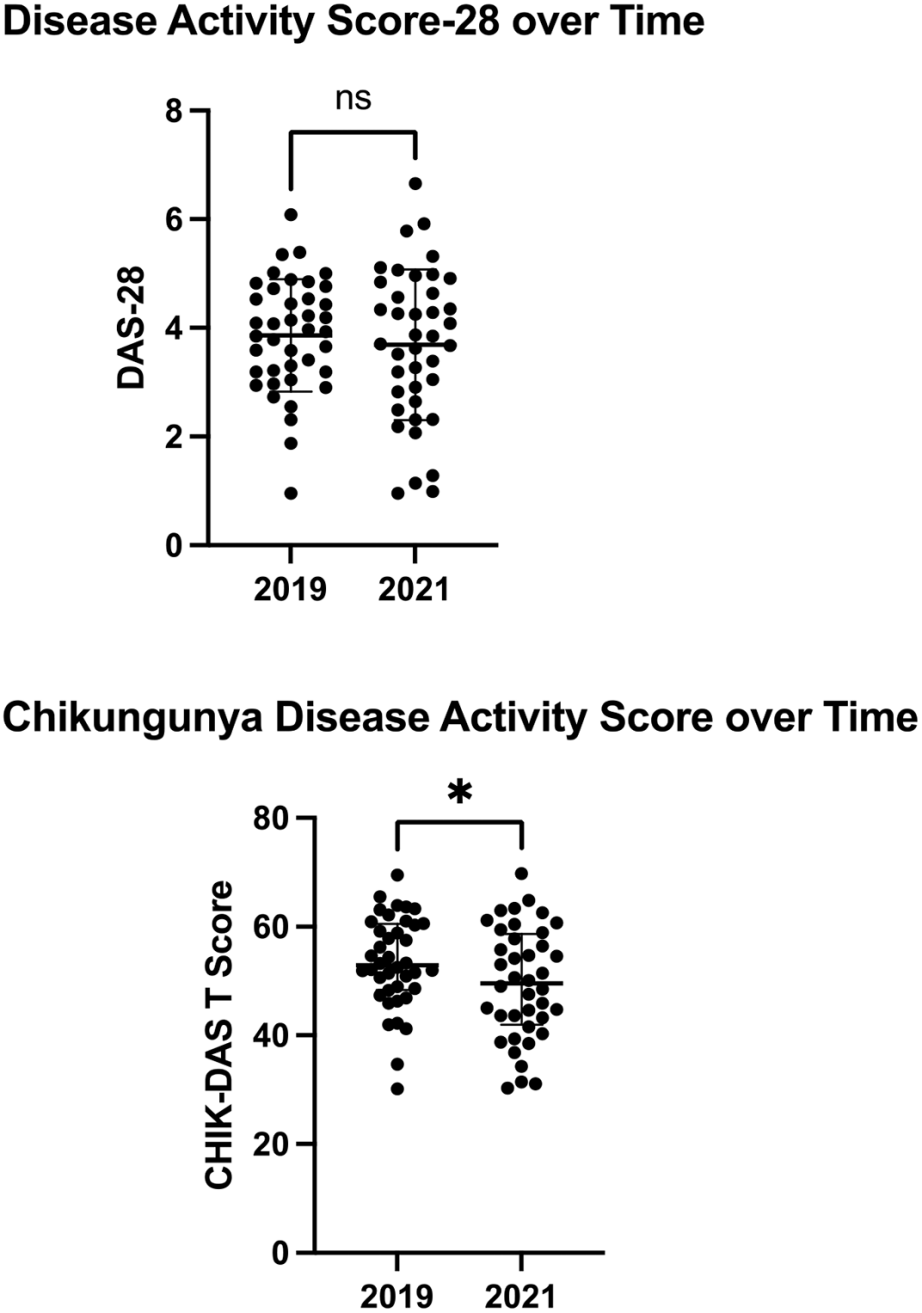
Disease activity over time shown by the Disease Activity Score-28 and Chikungunya arthritis disease activity score (CHIK-DAS) (n=40). Comparisons made by paired samples T-test. Horizontal bars indicate mean and standard deviation. *p<0.05. ns indicates non-significant.

Arthritis disease severity is shown via the DAS-28 and the CHIK-DAS in Figure 1. The participants had on average moderate disease severity as per DAS-28 (3.9 ± 1.0 in 2019 vs. 3.7 ± 1.4 in 2021, p=0.41). The CHIKDAS was more sensitive to change with a greater standardized effect size (Cohen’s d=0.41 compared to 0.14). The CHIKDAS improved by 3.9 T score-points from moderate to mild over 2 years (53.4 ± 8.4 in 2019 vs. 49.5 ± 10.3 in 2021, p=0.02).

**Figure 2.**
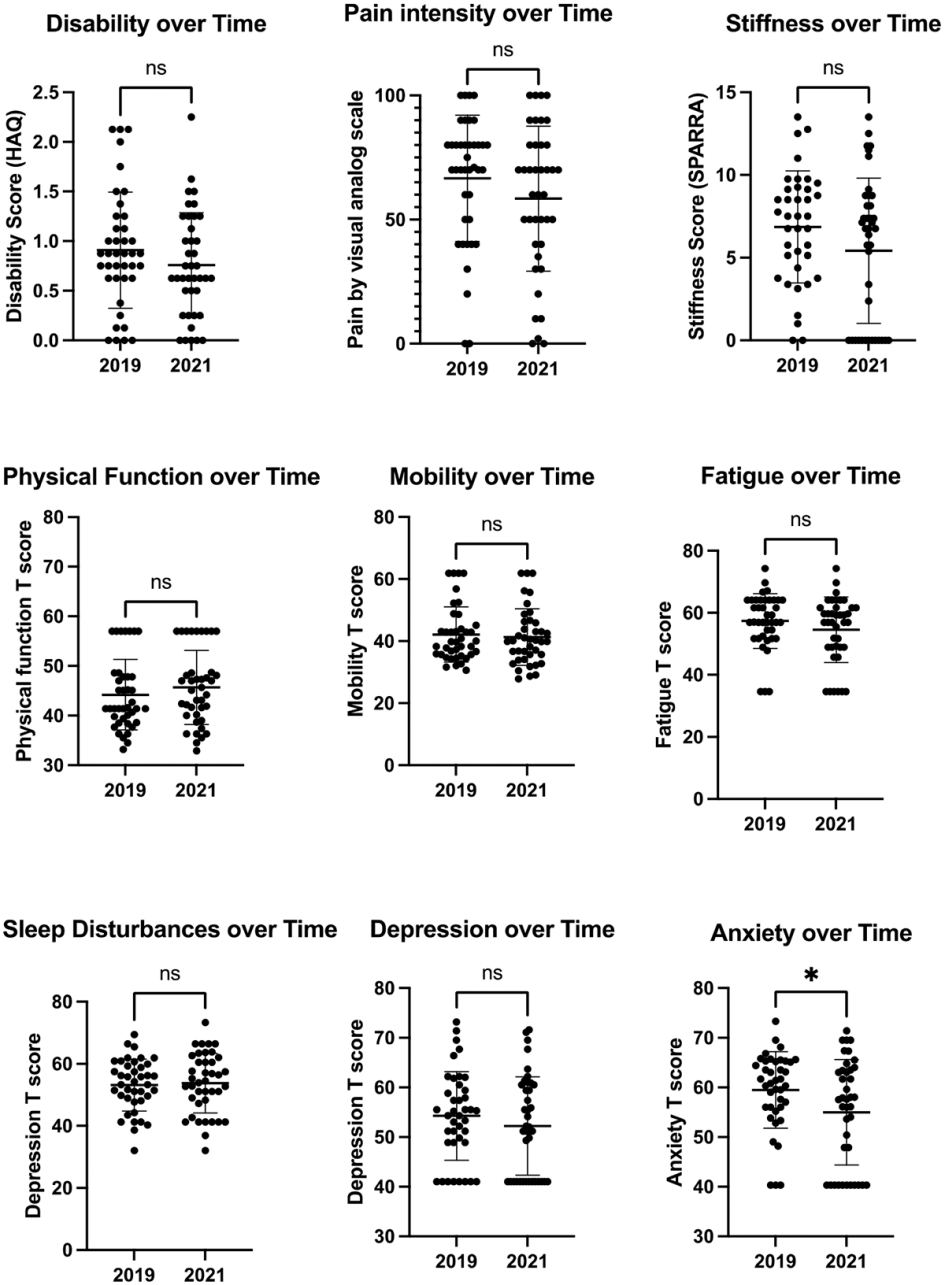
Patient-reported disability, pain, stiffness, physical function, mobility, fatigue, sleep disturbances, depression, and anxiety over time. Disability is measured by the Health Assessment Questionnaire. Pain was measured on a visual analog scale. Stiffness is measured by SPARRA stiffness score. Mobility, fatigue, sleep disturbances, depression and anxiety were measured via the Patient Reported Outcome Measurement Information System (PROMIS). Comparisons made by paired samples T-test. N=40 for all comparisons except for stiffness for which N=38. Horizontal bars indicate mean and standard deviation. *p<0.05. ns indicates non-significant.

Additional patient reported arthritis outcomes are shown in Figure 2. On average, the participants had moderate disability, pain, and stiffness that did not significantly change over time. A mild deficiency in physical function and mobility that did not change significantly over time was present. In addition, mild fatigue, sleep disturbances and depression that did not change significantly over time were also present. Anxiety decreased moderately over time (59.5 ± 7.7 in 2019 vs. 55.0 ± 10.6 in 2021, p=0.02).

### Joints affected over time

Affected joints are shown in Table 2. Joints were more commonly tender than swollen and the small joints were most affected (MCP, IFP and wrist). Joints affected did not change significantly over time.

### Arthritis medication use

Arthritis medication use in this population is shown in Table 3. The proportion of participants using various medications did not significantly change over time. The most utilized medications included acetaminophen and ibuprofen. Approximately one fourth used steroids or aspirin. No participants used methotrexate in this cohort.

### Arthritis flare precipitating factors

The precipitating factors for arthritis flares are shown in Table 4. Arthritis flares were precipitated by infection and exercise. Interestingly, a significantly greater proportion of participants reported exercise as a precipitating factor for arthritis flare two years later in the disease course (33% in 2019 vs. 73% in 2021, p=0.0004).

### T cell immunology and cytokines

**Figure 3.**
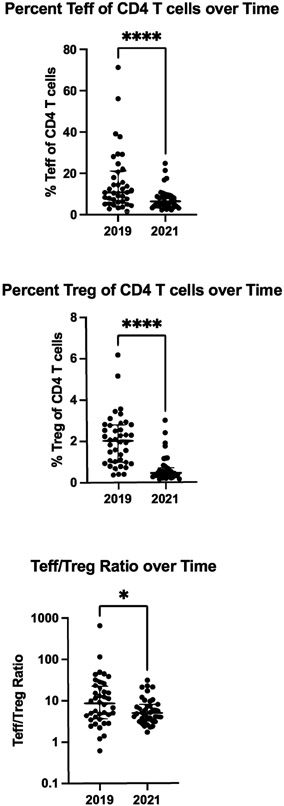
Effector T cells (Teff) and regulatory T cell (Treg) populations over time. Comparisons made by Wilcoxon signed-rank test. Horizontal bars indicate median and interquartile range. *p<0.05, **** p<0.0001.

**Figure 4.**
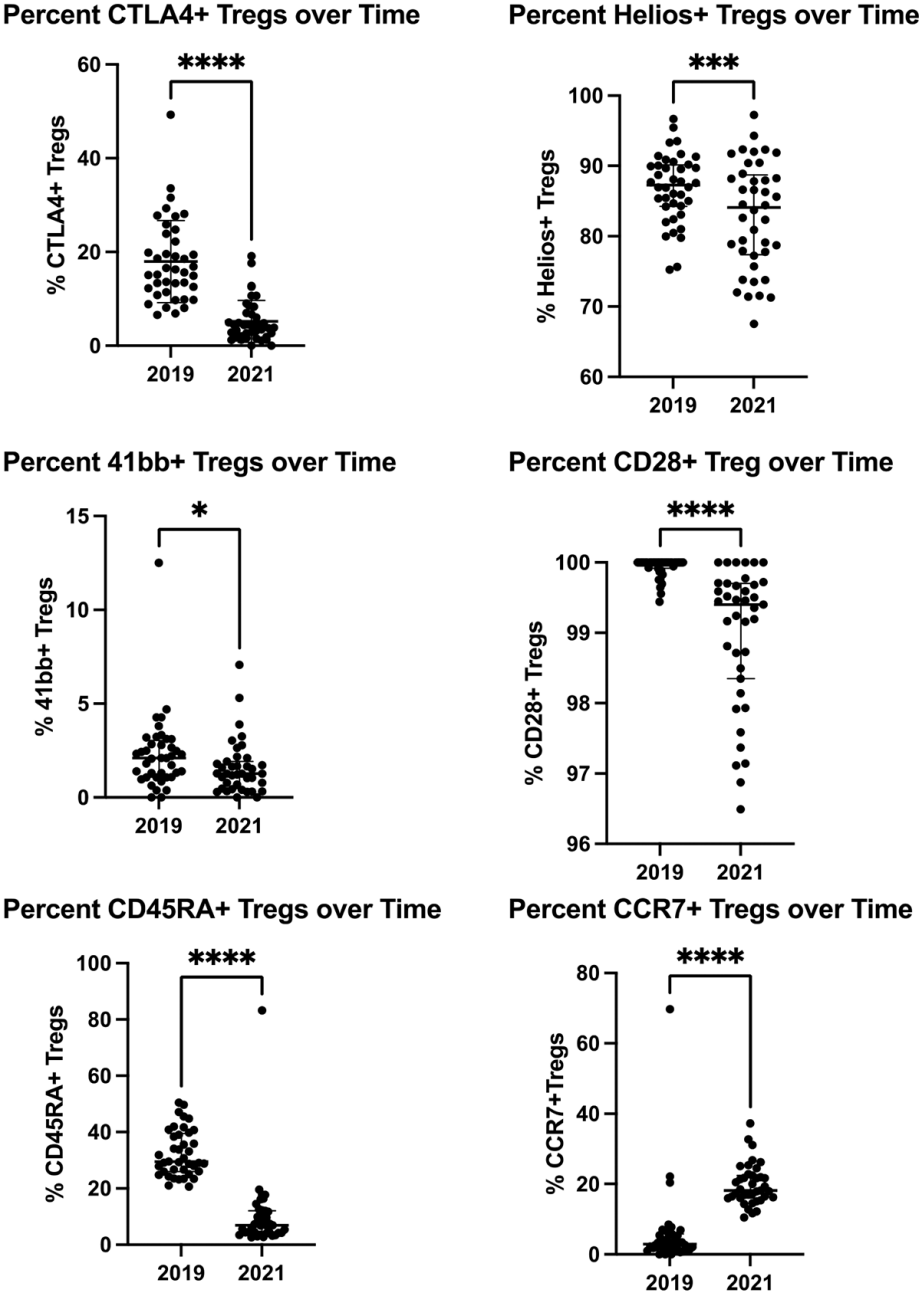
Regulatory T cell markers over time. Comparisons made by Wilcoxon signed-rank test. Horizontal bars indicate median and interquartile range. *p<0.05, ***p<0.001, ****p<0.0001.

Teff and Treg populations medians and interquartile ranges are shown in Figure 3. The percentage of both Teff and Treg of CD4^+^ T cells decreased over time (Teff Median (IQR) 11.3 (6.2-23.3) in 2019 to 6.4 (4.1-9.4) in 2021, p<0.0001; Treg Median (IQR) 1.58 (0.79-1.99) in 2019 to 1.04 (0.87-1.51) in 2021, p=0.004). Teff percentages decreased substantially, resulting in a Teff/Treg ratio approximately half the ratio two years later (Median (IQR) 8.57 (3.76-21.27) in 2019 to 4.96 (3.18-8.1) in 2021, p=0.03). Furthermore, markers of Treg immunosuppressive function that decreased over time are included in Figure 4 including CTLA4 (Median (IQR) 15.9 (11.8-23.1) in 2019 to 4.2 (1.9-6.8) in 2021, p<0.0001), Helios (Median (IQR) 87.3 (84.3-90.1) in 2019 to 84.1 (77.5-88.6) in 2021, p=0.0003), 41bb (Median (IQR) 2.1 (1.1-3.0) in 2019 to 1.3 (0.5-1.9) in 2021, p<0.029), Cd28 (Median (IQR) 100.0 (99.9-100.0) in 2019 to 99.4 (98.3-99.7) in 2021, p<0.0001), and CD45RA (Median (IQR) 29.4 (26.0-39.4) in 2019 to 6.9 (4.2-12.1) in 2021, p<0.0001). CCR7 expression increased over time (Median (IQR) 3.0 (1.4-5.7) in 2019 to 18.1 (16.0-22.4) in 2021, p<0.0001). Meanwhile, expression of HLA-DR was not significantly changed and therefore was not included in Figure 3 (Median (IQR) 38.2 (31.1-50.3) in 2019 to 43.0 (34.0-47.7) in 2021, p=0.56).

Cytokines levels over time are shown in Table 5. Peripheral blood cytokine levels (C-reactive Protein, IL-1β, IL-2, IL-4, IL-6, IL-10, IL-12p70, IL-17a, interferon-γ, and TNF) were not significantly changed over time.

Teff/Treg ratios were not correlated with chikungunya arthritis disease activity in either 2019 or 2021 (Table 6). Higher arthritis disease activity was positively correlated with CTLA4, CD28 and CD45RA and negatively correlated with CCR7 in 2019, which was a mean of about 4 years post-infection that was not conserved in 2021, which was a mean of about six-years post-infection.

## Discussion

To our knowledge this is the first study to follow patients four to six years after human chikungunya infection to understand the relationship between T cell immunology and arthritis disease activity over time. The study sample averaged nearly 4- and 6-years post-infection at the 2 study assessment times. The primary finding from this study was that rheumatologic symptoms persisted after CHIKV infection with mild to moderate intensity but that arthritis disease activity, Teff and Treg populations and activation markers waned over time.

These findings compliment what is already known about T cell immunology in CHIKV infection [13] where acute CHIKV infection leads to a CD8^+^ T cell predominance focused on killing virus infected cells via cytolytic mechanisms [14-17]. High initial antigen burden from high viral loads appear to cause continuous presentation of CHIKV epitopes leading to CD8^+^ T cell exhaustion demonstrating lower levels of functioning CD8^+^ T cells and poor efficiency clearing infected cells [16,18]. Then, CD4^+^ T cells predominate during the chronic phase producing inflammation and joint swelling in humans [17].

Our findings further add to the literature of the natural history of chronic chikungunya disease suggesting that over time, both CD4^+^ Teff and Treg populations wane in numbers and activation markers with a greater drop in CD4^+^ Teffs over time. It may be hypothesized that CD4^+^ Teff populations decrease over time as the remaining viral antigen is progressively cleared and correspondingly the need for Treg activation decreases as joint inflammation is subdued.

It is critical to highlight a key finding uncovered from this cohort data that in patients affected by mild to moderate arthritis after chikungunya infection, Treg activation markers progressively decrease and may be a therapeutic target. For example, we found decreases in markers of Treg immunosuppressive function (CTLA4) [19], Treg cell function (Helios and CD28) [20-22], Treg activation (HLA-DR^+^, CD45RA) [19], and Treg proliferation (4-1BB) [23]. We also found increases CCR7 expression that decreases Treg migration to inflamed tissue [21] where they are needed to control the inflammation. Furthermore, we found that Treg activation markers correlated with disease activity in 2019 that was not conserved two years later suggesting an evolution of the arthritis mechanisms over time. This data affirms another human study that demonstrated that a reduction in Tregs is associated with chronic arthritis and normalization of Tregs is associated with resolution of arthritis [24]. Therefore, based on this clinical study, Treg therapies may be a potential target for patients plagued by persistent arthritis for many years post infection.

Interleukin-2 [25] and interleukin-10 are important cytokines related to Treg proliferation and activation [24]. Our study suggests in this cohort with continued mild to moderate clinical arthritis that IL-2 and IL-10 levels are unchanged over time. These cytokines may also be a therapeutic target to drive patients with chronic arthritis after CHIKV infection into remission. Trials of low dose interleukin-2 are currently underway to study the efficacy of low-dose IL-2 stimulation of Tregs in autoimmune disease [26].

A final important finding from this study is the detailed characterization of the impact of arthritis in a cohort of patients an average of four years post infection. These patients exhibited persistent moderate disability, pain, and stiffness with mild fatigue, sleep disturbances and depression, and mild deficits in physical function and mobility. These findings highlight the persistent global impact on patient well-being years after initial infection. This information may inform health care strategies for the care of patients long after CHIKV epidemics.

### Limitations

There were several limitations in this study. Limited blood sample volume and sample size precluded measurement of additional T-cell markers and power to identify additional correlations. A larger sample size including additional regions with high CHIKV prevalence in future studies would increase generalizability. While statistical analyses to compare the characteristics of patients who were lost to follow up during the COVID-19 pandemic shown in Supplementary Tables 1-6 suggest that the patients who returned for follow up were similar in demographics and disease severity, anecdotally some of the patients who declined to return for a second visit reported their reason for lacking interest to return was improvement of their arthritis and therefore this cohort analysis may over-represent patients whose arthritis did not improve representing a selection bias. Another limitation is that time from infection to assessment varied across patients and the sample size was too small to allow reliably estimating curves for each parameter starting at, say, the immediate post-infection period. Lastly, multiple testing results in less certainty regarding the reported p values; however, this not a major concern because most of the significant results were strong with p-values <0.001 or better.

Furthermore, including a control group of individuals without CHIKV infection or arthritis and longer follow-up ranging from 5-10 years in future trials would allow for a more robust comparison and better understanding of the specific effects of CHIKV on T-cell immunology and arthritis over time. In addition, this study could benefit from a more detailed documentation and analysis of medication use, including dosages, duration, and potential effects on T-cell immunology and arthritis outcomes. Moreover, future studies should provide additional information with the inclusion of functional assessments such as grip strength or gait analysis to provide additional insights into the impact of persistent arthritis on physical function and disability. Lastly, future studies are needed to investigate the effectiveness of various treatment strategies, including targeted therapies based on the T-cell immunology findings, on disease progression and patient outcomes.

## Conclusions

In conclusion, this cohort study describes the T-cell immunology of a special group of patients with persistent arthritis an average of four years after infection with CHIKV. We found that the arthritis was still mild to moderate in intensity four to six years after initial infection with persistent effects on disability, pain, stiffness, fatigue, sleep disturbances, depression, physical function, and mobility. We found that both Treg and Teff numbers and functional markers decreased over time highlighting a potential therapeutic target for further research.

## Supplementary Material

JCI-24-191-Supplementary-File

## Figures and Tables

**Table 1.** Participant characteristics (N=40).

**Characteristic**	
Age in years mean (SD)	52.6 (14.5)
Percent female	85%
Percent secondary education or higher	55%
Months since infection mean (SD)	46.4 (16.5)

**Table 2.** Joints affected over time (N=40).

	2019	2021	
Scale	mean (SD)	mean (SD)	p^a^
Shoulder pain	0.75 (0.87)	0.73 (0.82)	0.8572
Elbow pain	0.48 (0.78)	0.55 (0.75)	0.7156
Wrist pain	1.00 (0.91)	0.75 (0.84)	0.1135
MCF pain	2.45 (3.36)	2.93 (3.47)	0.6492
IFP pain	2.73 (4.01)	3.15 (3.74)	0.3949
Knee pain	0.75 (0.93)	0.93 (0.83)	0.3743
Shoulder swelling	0.00 (0.00)	0.05 (0.22)	0.5000
Elbow swelling	0.03 (0.16)	0.10 (0.38)	0.5000
Wrist swelling	0.05 (0.32)	0.03 (0.16)	1.0000
MCF swelling	0.40 (1.08)	0.43 (0.96)	0.8458
IFP swelling	0.28 (0.88)	0.28 (0.64)	0.8184
Knee swelling	0.13 (0.40)	0.35 (0.66)	0.0664

a Wilcoxon Signed-Rank Test

**Table 3.** Medication use over time (N=40^a^).

Treatment	2019	2021	p^b^
Acetaminophen	92%	72%	0.0574
Ibuprofen	82%	70%	0.5078
Steroid	36%	18%	0.1460
Aspirin	26%	18%	0.7266
Methotrexate	0%	0%	1.0000
Other	18%	5%	0.0625

a N=38 or 39 for several items

b McNemar's Test

**Table 4.** Causes of arthritis relapse over time (N=40).

Cause of Pain	2019	2021	p^a^
Infection	23%	10%	0.2266
Exercise	33%	73%	0.0004
Medication	3%	0%	.
Other	10%	23%	0.2668

a McNemar's Test

**Table 5.** Cytokines over time (N=40^a^).

	2019		2021		
Laboratory Parameter	median	IQR	median	IQR	p^b^
C-Reactive Protein	1.50	(0.73 to 3.41)	0.45	(0.00 to 3.46)	0.1127
IL-6	1,648	(402 to 4,576)	1,382	(341 to 3,528)	0.0355
IL-10	245	(40 to 1131)	385	(70 to 838)	0.9020
IL-12p70	116	(94 to 655)	214	(94 to 579)	0.5045
IL-4	16.9	(16.9 to 43.3)	16.9	(16.9 to 40.8)	0.5046
TNFα	474	(145 to 1081)	458	(117 to 999)	0.2257
IL-2	53.0	(11.7 to 263.3)	46.5	(11.7 to 194.3)	0.4746
IL-1β	90.0	(90.0 to 132.4)	90.0	(90.0 to 118.3)	0.8599
IFNγ	441	(137 to 1447)	366	(85 to 1581)	0.7415
IL-17α	343	(87 to 1186)	290	(95 to 782)	0.5699

a N=39 for several parameters in 2021

b Wilcoxon Signed-Rank Test

**Table 6.** Spearman Correlations among select cytokines and CHIK-DAS for 2019^a^ and 2021^b^.

Variable	CHIK-DAS	Teff Treg ratio	% CTLA4 of Treg	% HELIOS of Treg	% CCR7 of Treg	% CD28 of Treg	% CD45a of Treg
CHIK-DAS	.	−0.07	0.17	−0.11	0.01	−0.01	−0.04
Teff / Treg ratio	0.00	.	0.01	0.11	0.17	−0.11	0.10
% CTLA4 of Treg	0.24**	0.20*	.	−0.23	0.01	0.47**	0.03
% HELIOS of Treg	0.01	0.10	0.19*	.	−0.30	−0.08	0.09
% CCR7 of Treg	−0.26***	−0.21**	−0.70***	−0.15	.	−0.38*	0.53***
% CD28 of Treg	0.27***	0.09	0.42***	0.01	−0.51***	.	−0.22
% CD45a of Treg	0.17*	−0.01	0.35***	0.02	−0.27***	0.30***	.

a Below diagonal (N=158)

b Above diagonal (N=39-40)

* p<.05

** p<.01

*** p<.001
