# Supplementary material for: The Natural History of Post-Chikungunya Viral Arthritis Disease Activity and T-cell Immunology: A Cohort Study
Source: J Cell Immunol. Author manuscript; Available in PMC 2024 Jun 13. (PMC11172407; doi:10.33696/immunology.6.191)
Supplement: JCI-24-191-Supplementary-File [file NIHMS1996538-supplement-JCI-24-191-Supplementary-File.pdf]

Supplement Table S1. Participant Characteristics in 2019 for those retained or lost to 2021 follow-up

	Sample		
Characteristic	Retained[a]	Lost to follow-up[b]	p[c]
Age mean (SD)	52.6 (14.5)	47.0 (17.0)	0.0674
Female	85%	82%	0.7979
Secondary education or higher	55%	70%	0.1501
Months since infection mean (SD)	46.4 (16.5)	50.1 (11.5)	0.2151

[a]N=40. Statistics are same as in main Table 1 for 2019.
[b]N=74
[c]Fisher's Exact Test or 2-sample T-test

Supplement Table S2. Arthritis outcomes in 2019 for those retained or lost to 2021 follow-up

	Sample				
	Retained[a]		Lost to follow-up[b]		
Characteristic	mean	SD	mean	SD	p[c]
DAS-28	3.9	(1.0)	3.8	(1.3)	0.6941
CHIK-DAS	53.4	(8.4)	51.8	(9.8)	0.3774
Disability measured by HAQ	0.9	(0.6)	0.7	(0.5)	0.0967
Pain Visual Analog Scale	68.5	(9.5)	66.2	(11.8)	0.2456
Stiffness measured by Sparra Score	6.9	(3.4)	5.6	(4.1)	0.1013
PROMIS Physical Function T-score	44.2	(7.1)	45.5	(8.6)	0.4055
PROMIS Fatigue T-score	57.4	(8.8)	53.7	(10.2)	0.0489
PROMIS Anxiety T-score	59.5	(7.7)	56.5	(8.0)	0.0566
PROMIS Sleep T-score	53.2	(8.4)	49.4	(8.8)	0.0285
PROMIS Depression T-score	54.3	(9.0)	52.0	(8.7)	0.1943
PROMIS Mobility T-score	42.1	(8.9)	43.6	(8.9)	0.4094

[a]N=40, except 2 missing SPARRA. Statistics are same as in main Table 2 for 2019.
[b]N=74, except 2 missing SPARRA.
[c]2-sample T-test

Supplement Table S3. Joints affected in 2019 for those retained or lost to 2021 follow-up

	Sample				
	Retained[a]		Lost to follow-up[b]		
Characteristic	mean	SD	mean	SD	p[c]
Shoulder pain	0.75	(0.87)	0.89	(0.94)	0.4775
Elbow pain	0.48	(0.78)	0.70	(0.89)	0.1832
Wrist pain	1.00	(0.91)	0.89	(0.94)	0.5350
MCF pain	2.45	(3.36)	1.88	(3.49)	0.1919
IFP pain	2.73	(4.01)	3.08	(4.11)	0.7139
Knee pain	0.75	(0.93)	0.95	(0.90)	0.2548
Shoulder swelling	0.00	(0.00)	0.00	(0.00)	1.0000
Elbow swelling	0.03	(0.16)	0.04	(0.26)	0.9488
Wrist swelling	0.05	(0.32)	0.22	(0.60)	0.0866
MCF swelling	0.40	(1.08)	0.43	(1.99)	0.0902
IFP swelling	0.28	(0.88)	1.16	(3.14)	0.9608
Knee swelling	0.13	(0.40)	0.24	(0.57)	0.2699

[a]N=40. Statistics are same as in main Table 3 for 2019.
[b]N=74
[c]Wilcoxon Rank-Sum Test

Supplement Table S4. Medication use in 2019 for those retained or lost to 2021 follow-up

	Sample		
Treatment	Retained[a]	Lost to follow-up[b]	p[c]
Acetaminophen	92%	92%	1.0000
Ibuprofen	82%	76%	0.6326
Steroid	36%	34%	0.8377
Aspirin	26%	24%	1.0000
Methotrexate	0%	1%	1.0000
Other	18%	15%	0.7878

[a]N ranges from 38 to 40. Statistics are same as in main Table 4 for 2019.
[b]N=74, except 2 missing Methotrexate.
[c]Fisher's Exact Test

Supplement Table S5. Causes of arthritis relapse in 2019 for those retained or lost to 2021 follow-up

	Sample		
Treatment	Retained[a]	Lost to follow-up[b]	p[c]
Infection	23%	23%	1.0000
Exercise	33%	35%	0.8381
Medication	3%	0%	0.3509
Other	10%	11%	1.0000

[a]N=40. Statistics are same as in main Table 5 for 2019.
[b]N=74
[c]Fisher's Exact Test

Supplement Table S6. T-cell subsets in 2019 for those retained or lost to 2021 follow-up

	Retained[a]		Lost to follow-up[b]		
Lab parameter	median	IQR	median	IQR	p[c]
% Treg of CD4 Tcells	1.58	(0.79 to 1.99)	1.38	(0.96 to 1.96)	0.8008
% Teff of CD4 Tcells	11.3	(6.2 to 23.3)	7.9	(4.6 to 18.1)	0.1323
% CTLA4 of Treg	15.9	(11.8 to 23.1)	18.2	(13.0 to 22.9)	0.4297
% HELIOS of Treg	87.3	(84.3 to 90.1)	90.8	(87.2 to 92.3)	0.0008
% HLADR of Treg	38.2	(31.1 to 50.3)	44.1	(38.9 to 52.6)	0.1806
% 41BB of Treg	2.1	(1.1 to 3.0)	2.2	(1.4 to 4.5)	0.1044
% CCR7 of Treg	3.0	(1.4 to 5.7)	2.2	(1.3 to 4.7)	0.2396
% CD28 of Treg	100.0	(99.9 to 100.0)	100.0	(100.0 to 100.0)	0.3787
% CD45a of Treg	29.4	(26.0 to 39.4)	28.0	(22.4 to 34.4)	0.0563
Teff / Treg ratio	8.57	(3.76 to 21.27)	6.62	(2.57 to 14.53)	0.2135
C-Reactive Protein	1.50	(0.73 to 3.41)	1.29	(0.47 to 3.46)	0.4401

[a]N=40. Statistics are same as in main Table 6 for 2019.
[b]N=74
[c]Wilcoxon Rank-Sum Test
